# Subcutaneous hydatid cysts occurring in the palm and the thigh: two case reports

**DOI:** 10.1186/1752-1947-2-273

**Published:** 2008-08-13

**Authors:** Abuzer Dirican, Bulent Unal, Cuneyt Kayaalp, Vedat Kirimlioglu

**Affiliations:** 1 Department of General Surgery, Medical Faculty of Inonu University, Malatya, Turkey

## Abstract

**Introduction:**

Hydatid cyst disease is common in some regions of the world and is usually located in the liver and lungs. This report presents two cases of primary hydatid cysts located subcutaneously: one in the medial thigh and one in the left palm between the index and middle fingers.

**Case presentations:**

A 64-year-old male farmer visited our hospital because a swelling on the right medial thigh had grown during the last year. Superficial ultrasound and computed tomography revealed a lesion resembling a hydatid cyst. A germinative membrane was encountered during surgical excision. Pathological examination was compatible with a hydatid cyst. The second case involved a 67-year-old male farmer who complained of a swelling that had grown in his left palm in the last year. The preliminary diagnosis was a lipoma. However, a hydatid cyst was diagnosed during surgical excision and after the pathological examination. The patient did not have a history of hydatid cyst disease and hydatid cysts were not detected in other organs. There has been no disease recurrence after following both patients for 3 years.

**Conclusion:**

A hydatid cyst should be considered in the differential diagnosis of subcutaneous cystic lesions in regions where hydatid cysts are endemic, and should be excised totally, with an intact wall, to avoid recurrence.

## Introduction

A hydatid cyst is a parasitosis caused by the larval form of *Echinococcus granulosus *or rarely *Echinococcus alveolaris*. The main hosts for *E*. *granulosus *are predators such as dogs, wolves, and foxes, while intermediate hosts include sheep, goats, and cattle. Humans are a coincidental intermediate host. The disease is more frequent in the Middle East, Central Europe, Australia, and South America, where the intermediate hosts are common. The organs affected most often are the liver (70%) and lungs (10–15%). Other locations are extremely rare [1]. Primary subcutaneous hydatid cyst is very rare and the incidence is unknown. In this report, we present two cases of primary hydatid cysts located subcutaneously: one in the medial thigh and one in the hand.

## Case presentations

**Figure 1:**
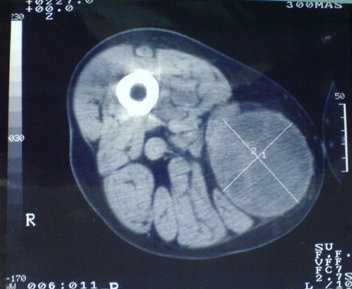
Subcutaneous hydatid cyst in the right medial thigh, displacing the muscles laterally.

**Figure 2:**
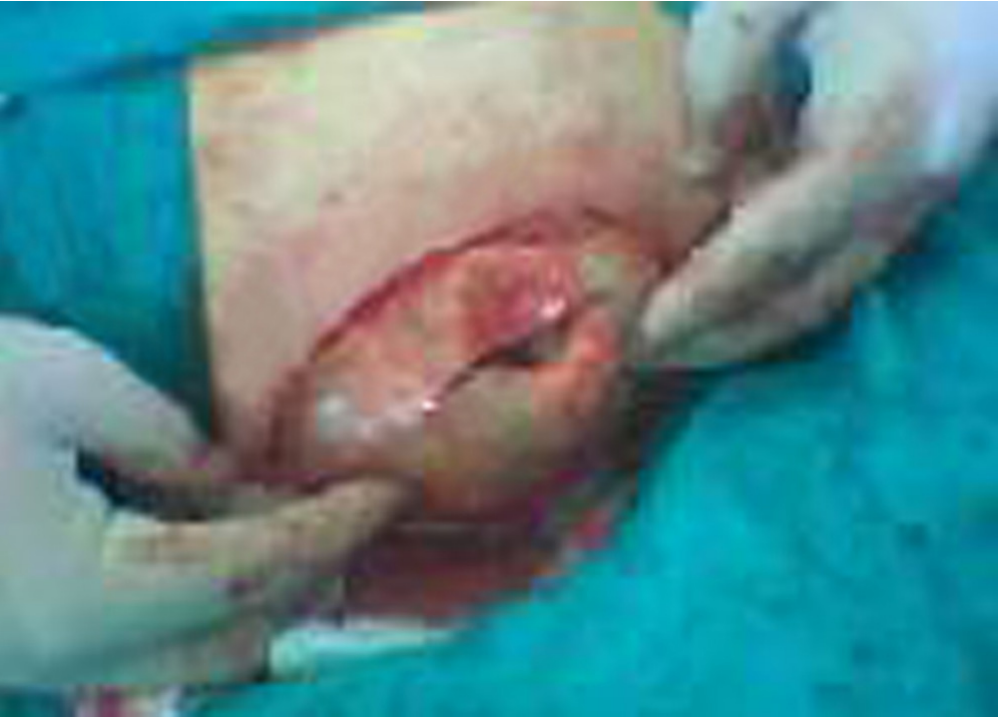
Subcutaneous hydatid cyst in the right medial thigh.

A 64-year-old male farmer visited our clinic because of a swelling on the medial thigh that had grown during the last year. On physical examination, a mobile, painless, fluctuant, 8 × 9 cm mass was palpated. The overlying skin was normal. The only abnormality in the pre-operative laboratory examination was an increased erythrocyte sedimentation rate (ESR 60 mm/hour). The patient had no history of surgery for a hydatid cyst in another organ. Ultrasound (US) and computed tomography (CT) showed a lesion resembling a hydatid cyst (Fig. 1). During surgical exploration under spinal anesthesia, the skin and subcutaneous layers were incised and the cyst was reached. Hypertonic saline (3% NaCl) was injected into the cyst and after waiting for 10 min, the cyst was completely excised. A germinative membrane was seen during excision (Fig. 2). We thought that the cyst was fertile as it contained daughter cysts. The surgical site was irrigated with 40% povidone iodine (Betadine^®^) and hypertonic saline. The subcutaneous layers and skin were closed in the standard manner.

Histopathological examination revealed a hydatid cyst, but no additional hydatid cysts were observed on US or CT of the abdomen and thorax; the indirect hemagglutination test for hydatid cysts was negative. The patient was started on albendazole for 3 months (15 mg/kg/day). No findings associated with local or systemic hydatid cysts were detected during a 3-year follow-up period.

**Figure 3:**
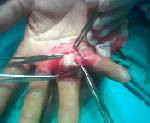
Germinative membrane of cyst localized in the palmar site of the hand.

The other case involved a 67-year-old male farmer who complained of a subcutaneous swelling inside the left palm between the index and middle fingers. Physical examination revealed a subcutaneous immobile 2 × 3 cm mass on the palmar side of the left hand between the thumb and index fingers. Surgical excision was planned with a pre-operative diagnosis of lipoma. A hydatid cyst was considered when a germinative membrane was seen during excision under local anesthesia (Fig. 3). We also thought that the cyst was fertile as it contained daughter cysts as in the previous patient. The cyst space was irrigated with 40% povidone iodine (Betadine^®^) and hypertonic saline. Total cyst excision and primary closure were performed, and histopathological examination revealed a hydatid cyst. The only abnormality in the pre-operative laboratory examination was an increased ESR (60 mm/hour). The patient had no history of surgery for a hydatid cyst in another organ, and no additional cysts were observed on US and CT of the abdomen and thorax. The indirect hemagglutination test for hydatid cysts was negative, and the patient was placed on albendazole for 3 months (15 mg/kg/day). No findings associated with local or systemic hydatid cysts were detected during a 3-year follow-up period.

## Discussion

Here we report two cases of primary subcutaneous hydatid cysts both treated surgically. In a large series, the distribution of hydatid cysts outside the liver and lungs was reported as 9% of cases [2]. Chevalier *et al*. reported that the incidence of subcutaneous hydatid cysts was 2%, but some of the patients had hydatid cysts in other organs too [3]. Subcutaneous hydatid cyst may be secondary or primary. In secondary cysts, there is a primary location of hydatid disease like liver, lung, or spleen that is operated or not operated. Reports of primary subcutaneous hydatid cysts are very rare [4-6], and we were unable to find a case of a palmar hydatid cyst in a literature review. In our cases, the hydatid cysts were located subcutaneously, the patients had not undergone previous surgery for hydatid cysts, and no hydatid cysts were found in other organs. Therefore, our patients were diagnosed as having primary subcutaneous hydatid cysts.

The mechanism of primary subcutaneous localization is unclear. After being ingested orally, under the action of gastric and intestinal enzymes, the oncosphere is released; it penetrates the intestinal wall, joins the portal system and reaches the liver. If the eggs attach to the liver, an hepatic hydatid cyst takes shape. Parasite eggs can pass to the systemic circulation and cause disease in other end organs. Larvae must pass through two filters (liver and lung) to form a solitary hydatid cyst, but that is very difficult. It is very possible that systemic dissemination via the lymphatic route accounts for cases with solitary cysts in uncommon sites [4]. Direct spread from adjacent sites may be another mechanism of infection provided a microrupture has occurred [7].

Diagnosing hydatid cysts is very difficult in patients living outside the endemic regions. Because exposure to the contents of the cyst can cause problems such as anaphylactic reaction and local recurrence, making the diagnosis pre-operatively is important. The diagnosis of a palmar hydatid cyst was not considered in our second patient pre-operatively since the mass was very small and this localization is very rare. When the cyst contents were seen during excision, the possibility of a hydatid cyst was then considered. No anaphylactic reaction developed in either patient.

The radiological findings of a thick cyst wall, calcification, daughter cysts, and a germinative membrane separate from the cyst wall are findings specific to hydatid cysts [8]. Our first case was diagnosed according to the appearance of the mass on superficial US and CT.

Serology is a useful tool for the diagnosis. The indirect hemagglutination (IHA) test is positive in more than 80% of liver hydatid cysts. However, false negative IHA results can be higher in other located hydatid cyst. In those cases, more specific serologic tests are mandatory. A positive indirect hemagglutination test for hydatid cysts is significant, although negative test results do not indicate the absence of the disease, as in our patients. Therefore, the most important diagnostic tool is the awareness of the physician, particularly for the unusual presentation of the disease.

The best treatment option is total surgical excision without opening the cyst. If the cyst cannot be excised without opening, the fluid contents should be removed, the laminated membrane should be totally excised, and the cyst pouch should be irrigated with protoscolicidal solutions [9]. Subcutaneous located cysts are more prone to rupture since they have not been diagnosed pre-operatively. We performed total cyst excision in both cases and irrigated the surgical areas with protoscolicidal agents. Identifying postoperative recurrence of the cyst in endemic regions is very difficult because the probability of formation of a new cyst is high. However, since our patients were still free of disease in the third postoperative year, any subsequent hydatid cyst formation may be considered to be a new infestation.

## Conclusion

Hydatid cyst should be considered in the differential diagnosis of subcutaneous cysts in regions where hydatid cysts are endemic. Total excision of the cyst with an intact wall is the best treatment.

## Competing interests

The authors declare that they have no competing interests.

## Authors' contributions

AD is the consultant surgeon who drafted the article and performed the operations. BU assisted in performing the surgery, took the pictures and helped revise the article. CK helped in acquisition of data and technical support. VK performed the literature search and helped in revision. All authors read, appraised and approved the final manuscript.

## Consent

Written informed consent was obtained from the patients before publication of this case series and any accompanying images. A copy of the written consent is available for review by the Editor-in-Chief of this journal.

## References

[B1] Kayaalp C Blumgart LH, Belghiti RJ, DeMatteo RP, Chapman WC, Büchler MW, Hann LE, D'Angleca M Hydatid cyst of the liver Surgery of the Liver, Biliary Tract, and Pancreas 2007 4 Philadelphia, PA: Saunders Elsevier 952 970

[B2] Prousalidis J Tzardioglou K Sgouradis L Katsohis C Aletras H Uncommon sites of hydatid disease World J Surg 1998 22 17 22 10.1007/s002689900343 9465756

[B3] Chevalier X Rhomouni A Bretagne S Martigny J Larget Piet B Hydatid cyst of the subcutaneous tissue without other involvement: MR imaging features AJR 1994 163 645 646 8079861 10.2214/ajr.163.3.8079861

[B4] Engin O Erdoğan M Solitary subcutaneous hydatid cyst Am J Trop Med Hyg 2000 62 583 584 11289668 10.4269/ajtmh.2000.62.583

[B5] Öztürk S Deveci M Yıldırım S Hydatid cyst in the soft tissue of the face without any primary Ann Plast Surg 2001 46 170 173 10.1097/00000637-200102000-00016 11216615

[B6] Ambo M Adachi K Okhawara A Postoperative alveolar hydatid disease with cutaneous involvement J Dermatol 1999 26 343 347 10405477 10.1111/j.1346-8138.1999.tb03485.x

[B7] Safioleas M Nikiteas N Stamatakos M Safioleas C Manti CH Revenas C Safioleas P Echinococcal cyst of the subcutaneous tissue: A rare case report Parasitol Int 2008 57 236 238 10.1016/j.parint.2007.11.002 18203655

[B8] Fikry T Harfaoui A Sibai H Zryoil BL Echinococcose musculaire primitive J Chir 1997 134 325 328 9772999

[B9] Duncan GJ Tooke SMT Echinococcus infestation of the biceps brachii Clin Orthop 1990 261 247 250 2245554

